# Resolving the Capacity‐Stability‐Cost Trilemma in Multi‐Principal‐Element Hydrogen Storage Alloys Through Multi‐Objective Optimization

**DOI:** 10.1002/advs.202513463

**Published:** 2025-08-31

**Authors:** Panpan Zhou, Qianwen Zhou, Wenzhe Liu, Nuo Lei, Yongpeng Chen, Jinghua Jiang, Dan Song, Hai‐Wen Li, Qin‐Yi Li, Lixin Chen, Xuezhang Xiao

**Affiliations:** ^1^ School of Advanced Energy Sun Yat‐Sen University Shenzhen Guangdong 518107 China; ^2^ State Key Laboratory of Silicon Materials School of Materials Science and Engineering Zhejiang University Hangzhou Zhejiang 310027 China; ^3^ Jiangsu Provincial Engineering Research Center for Structure‐Function Integrated Metallic Materials for Harsh Environments, College of Materials Science and Engineering Hohai University Changzhou Jiangsu 213200 China; ^4^ Department of Aeronautics and Astronautics Kyushu University Motooka744, Nishi‐Ku Fukuoka 819‐0395 Japan; ^5^ Key Laboratory of Hydrogen Storage and Transportation Technology of Zhejiang Province Hangzhou Zhejiang 310027 China

**Keywords:** cycling durability, de‐/hydrogenation properties, low‐pressure hydrogen storage, multi‐objective optimization, multi‐principal element Laves‐type alloys

## Abstract

Overcoming the capacity‐stability‐cost trilemma in hydrogen storage materials represents a fundamental Pareto‐type challenge for practical metal hydride applications. Current research efforts remain fragmented, typically pursuing single‐parameter optimization while lacking holistic approaches that concurrently satisfy all three criteria. Here, a novel design paradigm is proposed by orchestrating A/B‐side multi‐principal‐element alloys (MPEAs) in C14 Laves phases, enabling concurrent optimization of interstitial hydrogen storage environments and thermodynamics. Through three consecutive elemental screening and precise composition engineering, the optimized Ti_0.8_Zr_0.22_Mn_1.22_Cr_0.53_(VFe)_0.25_ MPEA achieves a breakthrough saturation capacity of 2.06 wt.% at 20 °C with merely 1.6 MPa electrolysis‐derived hydrogen pressure. More significantly, this material maintains an exceptional reversible capacity of 1.93 wt.% at 80 °C (against 0.1 MPa back pressure), achieving an 93.7% capacity utilization efficiency. Such outstanding performance under limited operating conditions surpasses the vast majority of known C14 Laves‐phase materials. Equally noteworthy is the superior structural‐property robustness enabled by local strain release through timely pulverization during repeated hydrogen insertion/extraction, which results in negligible influence in hydrogen storage properties, crystallographic structure, or elemental distribution throughout extended cycling. These findings establish new design guidelines for high‐capacity, long‐cycle‐life, low‐cost hydrogen storage materials operating under energy‐efficient conditions.

## Graphical abstract

This work presents a paradigm‐shifting multi‐objective optimization strategy for hydrogen storage materials, simultaneously addressing the intrinsic capacity‐stability‐cost trade‐off through precisely engineered Ti‐Zr‐Mn‐Cr‐VFe multi‐principal‐element alloys in C14 Laves phases.

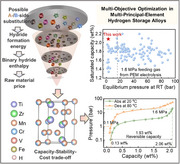

## 1 Introduction

The global energy transition demands breakthrough solutions to simultaneously address the trilemma of energy security, environmental sustainability, and economic viability.^[^ ^1^, ^2^, ^3^, ^4^ ^]^ Within this paradigm, hydrogen energy has emerged as a particularly compelling solution, distinguished by its abundant availability, zero‐emission energy conversion process, and exceptional high energy density ^[^ ^5^, ^6^ ^]^ A crucial technological barrier hindering large‐scale hydrogen application remains the development of storage and delivery systems that concurrently satisfy demanding density specifications and strict safety standards ^[^ ^7^, ^8^, ^9^ ^]^.In this context, solid‐state hydrogen storage materials (HSMs) have gained considerable prominence owing to their distinctive temperature‐pressure characteristics and reversible de‐/hydrogenationproperties.^[^ ^10^, ^11^, ^12^, ^13^, ^14^, ^15^, ^16^ ^]^

Within the investigated hydrogen storage systems, AB_2_‐type Ti‐based HSMs demonstrate prominent advantages, including high gravimetric hydrogen storage density (1.80 wt.%) and superior room‐temperature de‐/hydrogenationproperties.^[^ ^17^, ^18^, ^19^, ^20^, ^21^ ^]^ Multi‐principal element alloys (MPEAs) enable synergistic optimization of hydrogen storage properties through precise configuration engineering, including compositional adjustment of elemental species and their concentrations.^[^ ^22^, ^23^, ^24^, ^25^ ^]^ For example, Tu et al.^[^ ^26^ ^]^ developed (Ti_0.8_Z_r0.2_)_1.1_Mn_1.2_Cr_0.55_Ni_0.2_V_0.05_ MPEA for fuel‐cell bike. It exhibits a hydrogenation equilibrium pressure of 1.07 MPa at 25 °C, and can reach a saturation hydrogen storage capacity of 1.82 wt.% at 4.0 MPa. Similarly, (TiZr_0.1_)_1.1_Cr_1.5_Fe_0.2_Mn_0.3_ MPEA optimized by Li et al.^[^ ^27^ ^]^ demonstrates a hydrogenation equilibrium pressure of 1.56 MPa at 20 °C, yet requires nearly 10 MPa to achieve full saturation at its maximum 2.0 wt.% capacity. Proton‐exchange membrane (PEM) electrolysis, as the prevailing technology for green hydrogen production, delivers hydrogen at an intrinsic 1.6 MPa output pressure. However, the operating pressure requirements of current‐generation AB_2_‐type MPEAs substantially exceeds the PEM benchmark. This pressure mismatch necessitates additional energy‐intensive compression steps when utilizing electrolytically generated hydrogen, thereby compromising the overall energy efficiency of the hydrogen storage and delivery system ^[^ ^28^, ^29^ ^]^ Besides, prior reports predominantly address individual aspects of hydrogen storage capacity, cycling stability, or cost‐effectiveness, with no systematic investigation simultaneously encompassing all three parameters. To advance practical applications, there is an urgent need for rational design of novel low‐cost MPEAs to enable reversible de‐/hydrogenation under mild conditions through precise elemental engineering.

In this work, we first screened potential A/B‐side substitution elements for TiMn_2_ alloy from common transition metals based on key indicators such as hydride formation energy, hydrogen binding capability, and cost. Subsequently, we conducted systematic elemental substitutions and investigated their effects and underlying mechanisms on the crystallographic structure and hydrogen storage properties. Through comprehensive evaluation under practical operating conditions (room‐temperature, ≤1.6 MPa hydrogen pressure), Ti_0.8_Zr_0.22_Mn_1.22_Cr_0.53_(VFe)_0.25_ MPEA was selected as the optimal candidate. Afterward, this MPEA underwent systematic comparative analysis and structural‐property stability assessment. Our findings establish new design guideline for next‐generation MPEAs capable of efficient operation under energy‐saving conditions.

## 2 Results and Discussion

### 2.1 Elemental Scanning

**Figure 1:**
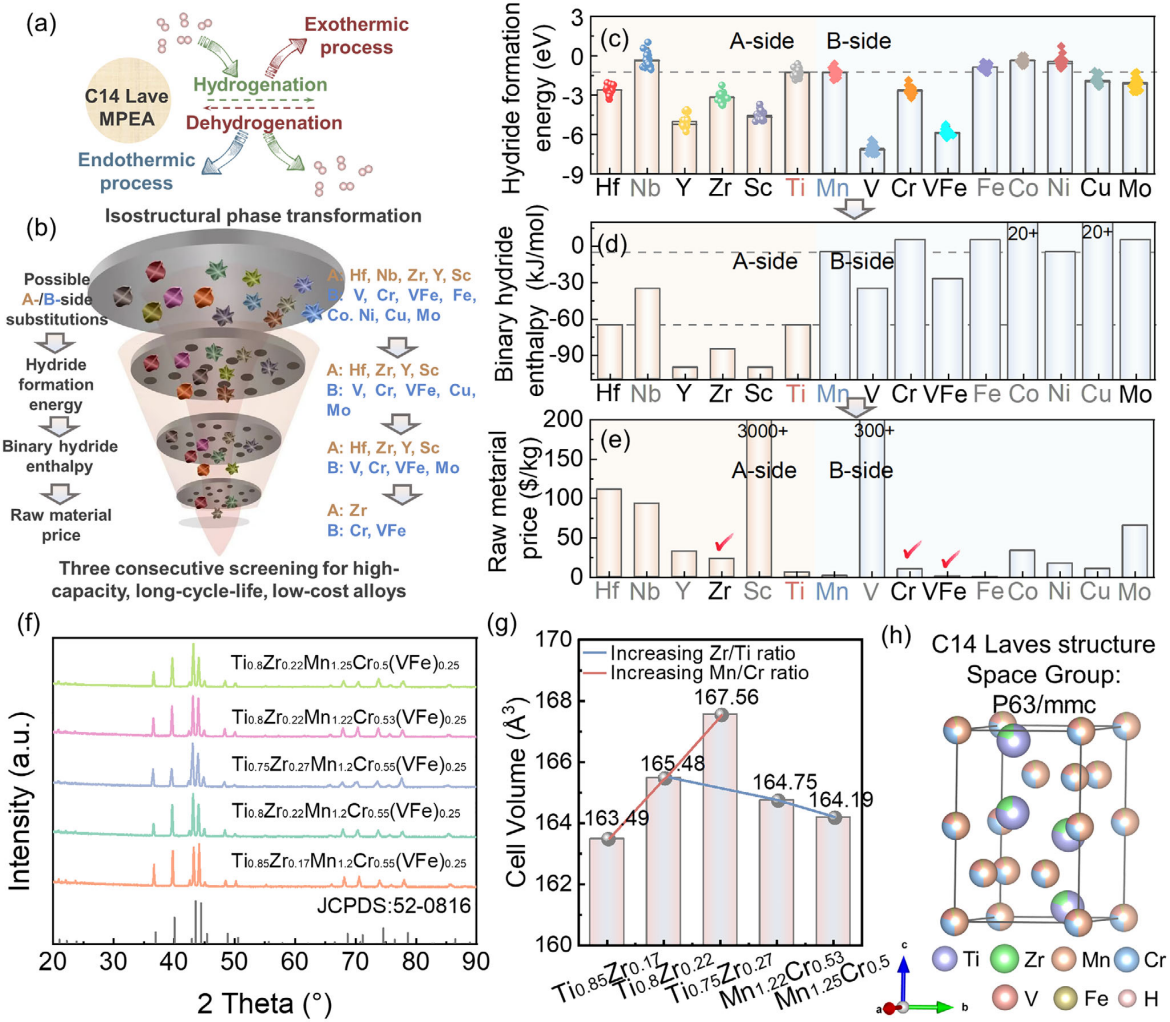
Schematic diagram of a) isostructural phase transformation of C14 Laves‐type MPEAs and b) three consecutive screening for high‐capacity, long‐cycle‐life, low‐cost alloys. c) Hydride formation energy of *AB*_2_H_3_. d) Binary hydride enthalpy and e) raw material price of related elements. f) XRD patterns, g) cell volume, and h) crystallographic structure of Ti‐Zr‐Mn‐Cr‐(VFe)‐based MPEAs.

TiMn_2_ is the most typical and fundamental C14‐type HSM. However, pure Ti‐Mn HSM are prone to irreversible TiH phase formation during hydrogen cycling, adversely affecting their structural stability.^[^ ^30^ ^]^ Consequently, amplifying the isostructural phase transformation driving force emerges as a crucial descriptor for cycling stability (**Figure** **1**a,b). Potential A‐side (Ti) substitutions include Hf, Nb, Y, Zr, and Sc, while B‐side (Mn) substitutions comprise V, Cr, Fe, Co, Ni, Cu, and Mo. A lower hydride formation energy indicates a stronger driving force for hydrogenation, meaning the alloy is more likely to undergo hydrogenation under limited hydrogenation conditions. Accordingly, the resulting hydride exhibits higher thermodynamic stability and structural stability.^[^ ^22^, ^23^ ^]^ As shown In Figure 1c, hydride formation energy (Δ*E* = *E*(*A*_4_ *B*_8_H_12_)‐*E*(*A*_4_ *B*_8_)‐6*E(H_2_)) reveals that A‐side substitutions with Hf, Y, Zr, or Sc yield Δ*E* values (−2.6261 to −5.0414 eV) lower than TiMn_2_ (−1.2734 eV), attributed to their larger atomic radii and lower electronegativity. Generally, when an alloy is substituted with elements of larger atomic radius, both its unit cell size and hydrogen storage interstitial sites expand, facilitating hydrogen atom insertion and storage. Moreover, lower electronegativity typically correlates with higher hydrogen affinity, enabling the element to enhance the alloy's valence electron transfer capability. This strengthens the metal‐hydrogen bonding interaction, thereby improving the material's thermodynamic stability.^[^ ^24^, ^31^, ^32^, ^33^, ^34^, ^35^, ^36^, ^37^ ^]^ B‐side substitutions that V, Cr, and Mo can significantly improve thermodynamic stability, where V incorporation achieves a remarkably low Δ*E* of −7.1333 eV, outperforming all A‐side variants.

Besides, binary hydride formation enthalpy (Δ*H*_f_) is the key metric for assessing the hydrogen affinity of elements, where increasingly positive values directly reflect both enhanced hydrogen binding difficulty and diminished storage capacity. Subsequently, as displayed in Figure 1d, Cu is excluded due to its significantly more positive Δ*H*_f_ (> 20 kJ mol^−1^). Besides, the raw material cost is another critical factor for practical engineering applications. As shown in Figure 1e, Zr emerges as the most cost‐effective substitution element for A‐side substitution. For the B‐side, while pure V commands an extremely high price (> 300 $ kg^−1^), VFe alloy (V80Fe) proves significantly more economical at just 1.13 $ kg^−1^, which is even lower than Mn (1.87 $ kg^−1^). After systematically evaluating the stability‐capacity‐cost triangle, Zr (A‐side), Cr (B‐side), and VFe alloy (B‐side) emerge as optimal substituents for TiMn_2_, to simultaneously address cycling durability, storage performance, and cost considerations.

### 2.2 Phase Structure

Based on the elemental screening results and solid solubility considerations, Ti_0.85‐_ *_x_* Zr_0.17+_ *_x_* Mn_1.2_Cr_0.55_(VFe)_0.25_ (*x* = 0, 0.05, 0.10) and Ti_0.80_Zr_0.22_Mn_1.2+_ *_y_* Cr_0.55‐_ *_y_* (VFe)_0.25_ (*y* = 0, 0.02, 0.05) MPEAs were designed. XRD patterns and corresponding crystallographic parameters are systematically presented in Figure 1f and Table S1 (Supporting Information), respectively. These diffraction patterns exhibit perfect indexing to the hexagonal C14 Laves phase (JCPDS: 52‐0816), with no detectable secondary phases, confirming the successful formation of phase‐pure C14 Laves structures. The stabilization of C14 Laves phase in these MPEAs is governed by two critical factors: the atomic size ratio (*r*_A_/*r*_B_) and the average number of outer electrons (ANOE).^[^ ^38^ ^]^ For all MPEAs investigated, ANOE value (5.868–5.703) falls within the characteristic range of 5.4–7.0 required for C14 phase formation. Besides, the calculated *r*_A_/*r*_B_ ratios ranging from 1.161 to 1.171 perfectly satisfy the geometric criterion (1.05–1.68) for C14 structure stabilization. Progressive substitution of Ti (147 pm) with larger Zr (160 pm) atoms in Ti_0.85‐_ *_x_* Zr_0.17+_ *_x_* Mn_1.2_Cr_0.55_(VFe)_0.25_ (*x* = 0, 0.05, 0.10) MPEAs induces a systematic expansion of crystallographic parameters (Figure 1g; Table S1, Supporting Information), with the unit cell volume increasing isotopically from 163.49 Å^3^ (*a* = 4.8331 Å, *c* = 8.0819 Å) to 167.56 Å^3^ (*a* = 4.8469 Å, *c* = 8.2358 Å). Conversely, in Ti_0.80_Zr_0.22_Mn_1.2+_ *_y_* Cr_0.55‐_ *_y_* (VFe)_0.25_ (*y* = 0, 0.02, 0.05) MPEAs, partial substitution of Cr (130 pm) by smaller Mn (127 pm) at the B‐side resulted in isotropic lattice contraction (Table S1, Supporting Information). Both substitution series are in full accordance with the classical law of elemental substitution on crystallographic parameters.^[^ ^39^, ^40^ ^]^ Furthermore, taking Ti_0.8_Zr_0.22_Mn_1.22_Cr_0.53_(VFe)_0.25_ MPEA as an example, ICP analysis reveals that the actual contents of Ti, Zr, Mn, Cr, V, and Fe are in excellent agreement with the theoretically designed composition, with a maximum deviation of no more than 1.8% (Table S2, Supporting Information). This result further confirms that ILM offers outstanding precision and controllability in regulating the composition of MPEAs. Complementary SEM and EDX mapping analyses (Figure S1, Supporting Information) confirm the formation of a single‐phase C14 Laves structure with satisfactory elemental homogeneity in all ILM‐processed Ti‐Zr‐Mn‐Cr‐(VFe) MPEAs in this work.

### 2.3 Hydrogen Storage Properties

**Figure 2:**
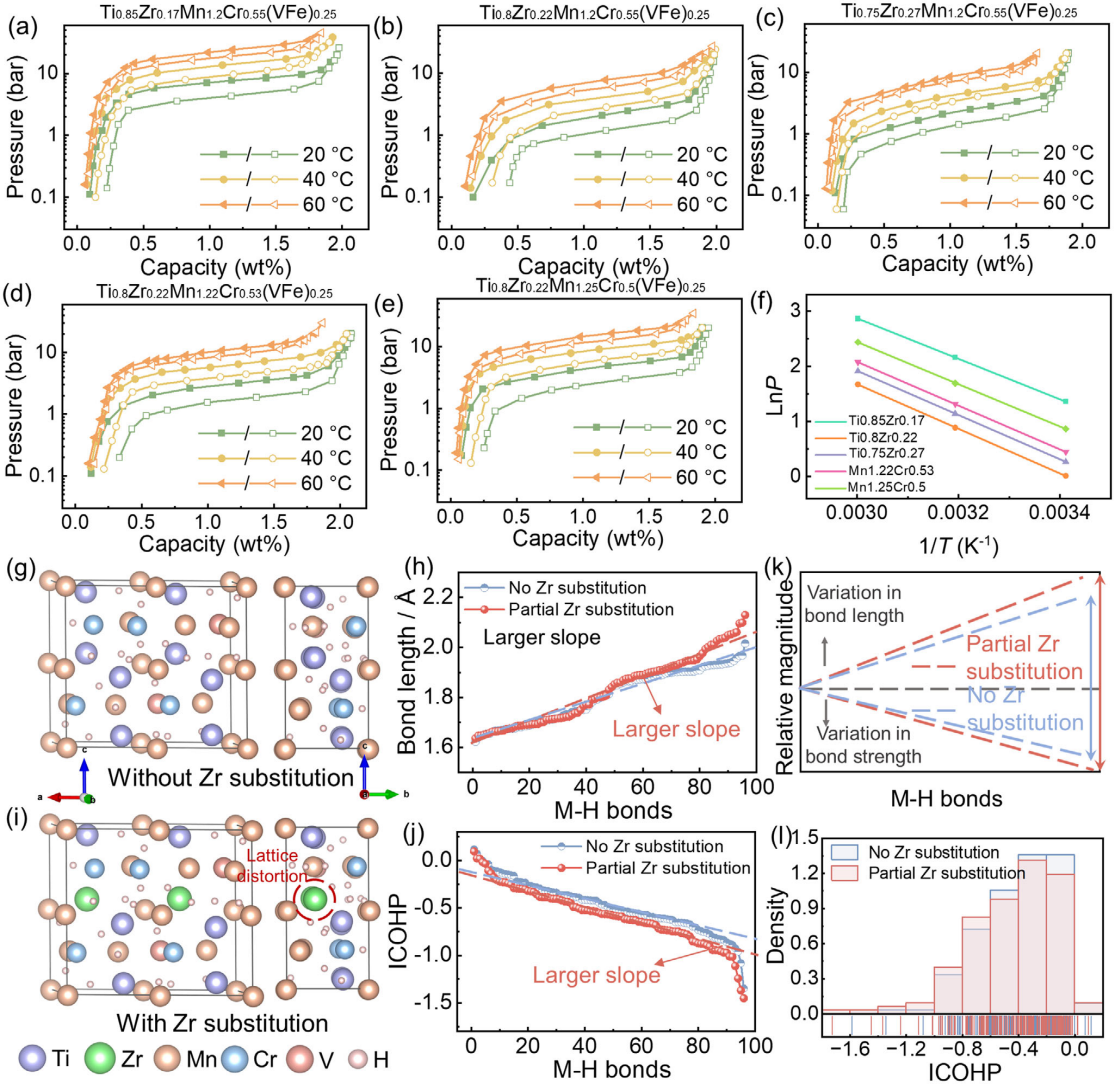
PCT curves of a) Ti_0.85_Zr_0.17_Mn_1.2_Cr_0.55_(VFe)_0.25_; b) Ti_0.80_Zr_0.22_Mn_1.2_Cr_0.55_(VFe)_0.25_; c) Ti_0.75_Zr_0.27_Mn_1.2_Cr_0.55_(VFe)_0.25_; d) Ti_0.80_Zr_0.22_Mn_1.22_Cr_0.53_(VFe)_0.25_; e) Ti_0.80_Zr_0.22_Mn_1.25_Cr_0.5_(VFe)_0.25_ MPEAs and f) related dehydrogenation Van't Hoff equation fitting. Optimized geometrical configurations of MPEA: g) pristine structure (Zr‐free) and i) partial Zr‐substituted structure, both relaxed under the energy minimization criterion. h) Bond lengths, j) ICOHP results, and k) comprehensive illustration of the M–H bonds. l) Distribution of ICOHP values with or without Zr substitution.

Subsequently, the systematic effects of A/B‐side elemental optimization on hydrogen storage properties in Ti‐Zr‐Mn‐Cr‐(VFe) MPEAs were investigated. While conventional crystallographic principles suggest that increasing Zr content (with larger atomic radius) should expand the unit cell and consequently enhance hydride formation, manifesting as lower de‐/hydrogenation equilibrium pressures and higher hydrogen capacities. However, Ti_0.85‐_ *_x_* Zr_0.17+_ *_x_* Mn_1.2_Cr_0.55_(VFe)_0.25_ (*x* = 0, 0.05, 0.10) MPEAs exhibits unexpected non‐monotonic behavior (**Figure** **2**a–c and **Table** **1**). The dehydrogenation equilibrium pressure initially decreases from 3.93 bar (*x* = 0) to 1.02 bar (*x* = 0.05), then increases to 1.32 bar (*x* = 0.10). Similarly, the room‐temperature saturated capacity at 1.6 MPa first rises from 1.92 to 1.97 wt.% before declining to 1.89 wt.%. More notably, the plateau slope factor (*S*_f_) demonstrates significant Zr‐dependence, increasing from 0.285 (*x* = 0) to 0.476 (*x* = 0.05) and 0.671 (*x* = 0.10), ultimately impairing complete hydrogenation under practical pressure conditions. The aforementioned abnormal behavior primarily stems from the larger atomic radius and stronger hydrogen affinity of Zr.^[^ ^41^ ^]^

**Table 1:** Hydrogen storage properties of Ti‐Zr‐Mn‐Cr‐(VFe)‐based MPEAs.

Alloy	20 °C			60 °C			Δ*H*_d_ ^d)^ [kJ∙mol^−1^]	Saturated capacity^f)^ [wt.%]
	*P*_a_ ^a)^ [bar]	*P*_d_ ^b)^ [bar]	*H*_f_ ^c)^	*P*_a_ ^a)^ [bar]	*P*_d_ ^b)^ [bar]	*H*_f_ ^c)^		
Ti_0.85_Zr_0.17_Mn_1.2_Cr_0.55_(VFe)_0.25_	7.06	3.93	0.59	22.16	17.71	0.22	30.56	1.92
Ti_0.8_Zr_0.22_Mn_1.2_Cr_0.55_(VFe)_0.25_	1.85	1.02	0.59	6.74	5.37	0.23	33.67	1.97
Ti_0.75_Zr_0.27_Mn_1.2_Cr_0.55_(VFe)_0.25_	2.21	1.32	0.52	8.25	6.84	0.19	33.39	1.89
Ti_0.8_Zr_0.22_Mn_1.22_Cr_0.53_(VFe)_0.25_	2.82	1.55	0.60	10.03	8.13	0.21	33.04	2.06
Ti_0.8_Zr_0.22_Mn_1.25_Cr_0.5_(VFe)_0.25_	4.36	2.40	0.60	14.48	11.57	0.22	31.89	1.90

^a)^ *P*_a_ is the midpoint pressure of the related hydrogenation PCT curve;

^b)^ *P*_d_ is the midpoint pressure of the related dehydrogenation PCT curve;

^c)^ *H*_f_ refers to the equilibrium pressure hysteresis that is determined by the equation of *H*_f_ = ln (*P*_a_/*P*_d_);

^d)^ Δ*H*_d_ is the dehydrogenation enthalpy change calculated by Van't Hoff equation;

^f)^ Saturated capacity is the maximum hydrogen storage capacity at 20 °C and 1.6 MPa hydrogen.

To investigate the effect of Zr substitution on the structure and hydrogen storage properties of the MPEA, theoretical calculations were conducted (Figure 2g–l). Considering that in the optimized MPEA system, the stoichiometry of VFe on the B side is only 0.25, and within VFe, the mass fraction of Fe is 0.2, the number of Fe atoms in the 2 × 1 × 1 HCP‐type A_4_B_8_ supercell is only ≈0.4. Therefore, the Fe element was not considered. As evidenced in Figure 2g,i and Figure S2 (Supporting Information), the substitution of larger‐radius Zr atoms at the A‐side induces pronounced atomic crowding and localized lattice distortion. Afterward, metal‐hydrogen bond length distributions and integrated crystal orbital Hamilton population (ICOHP) value were analyzed. Comparative results reveal that the Ti_6_Zr_2_Mn_10_Cr_4_V_2_H_24_ system exhibits steeper gradients of bond length and ICOHP value of metal‐hydrogen bonds (bond length within 1.5–2.5 Å) than its Ti_8_Mn_10_Cr_4_V_2_H_24_ counterpart (Figure 2h,j,k). As shown in Figure 2j,l, the ICOHP value distribution of Zr‐substituted alloys shifts toward more negative values, indicating stronger metal‐hydrogen bond strength, which aligns well with the experimentally observed lower de‐/hydrogenation equilibrium pressures. These findings demonstrate that Zr incorporation not only geometrically complicates interstitial sites through lattice strain but also electronically tailors the hydrogen binding energy landscape, ultimately contributing to the substantial enhancement in the plateau slope.

Among the Ti_0.85‐_ *_x_* Zr_0.17+_ *_x_* Mn_1.2_Cr_0.55_(VFe)_0.25_ (*x* = 0, 0.05, 0.10) MPEAs, the Ti_0.85_Zr_0.17_Mn_1.2_Cr_0.55_(VFe)_0.25_ MPEA demonstrates superior comprehensive hydrogen storage properties, exhibiting a plateau slope of 0.476 and saturated hydrogen storage capacity of 1.97 wt.% at 20 °C and 1.6 MPa hydrogen pressure. To further enhance the hydrogen storage capability, we strategically engineered the B‐side composition by increasing Mn content in Ti_0.80_Zr_0.22_Mn_1.2+_ *_y_* Cr_0.55‐_ *_y_* (VFe)_0.25_ (*y* = 0, 0.02, 0.05) MPEAs. Figure 2b,d,e and Table 1 demonstrate that elevating the Mn content from 1.2 to 1.25 leads to increasing hydrogenation equilibrium pressure from 1.85 to 4.36 bar while maintaining almost consistent plateau slopes ≈0.4. The saturated hydrogen storage capacity exhibits a non‐monotonic trend, initially rising from 1.89 wt.% at *y* = 0 to a maximum of 2.06 wt.% at *y* = 0.02 before decreasing slightly to 1.90 wt.% at *y* = 0.05. This pressure elevation aligns with Lundin's theory,^[^ ^31^ ^]^ where the observed unit cell contraction correlates with reduced interstitial site sizes and consequently higher hydrogenation energy barrier and hydrogenation equilibrium pressure. The beneficial role of Mn in boosting hydrogen storage capability is counterbalanced by its pressure‐elevating effect, creating an optimization conflict that manifests as the non‐linear composition‐capacity relationship observed in these MPEAs (Table 1). Furthermore, these MPEAs systematically follow the fundamental thermodynamic correlation where decreased dehydrogenation enthalpies correspond to elevated equilibrium pressures, consistent with previous reported rules and thermodynamic law.^[^ ^34^, ^42^, ^43^ ^]^

**Figure 3:**
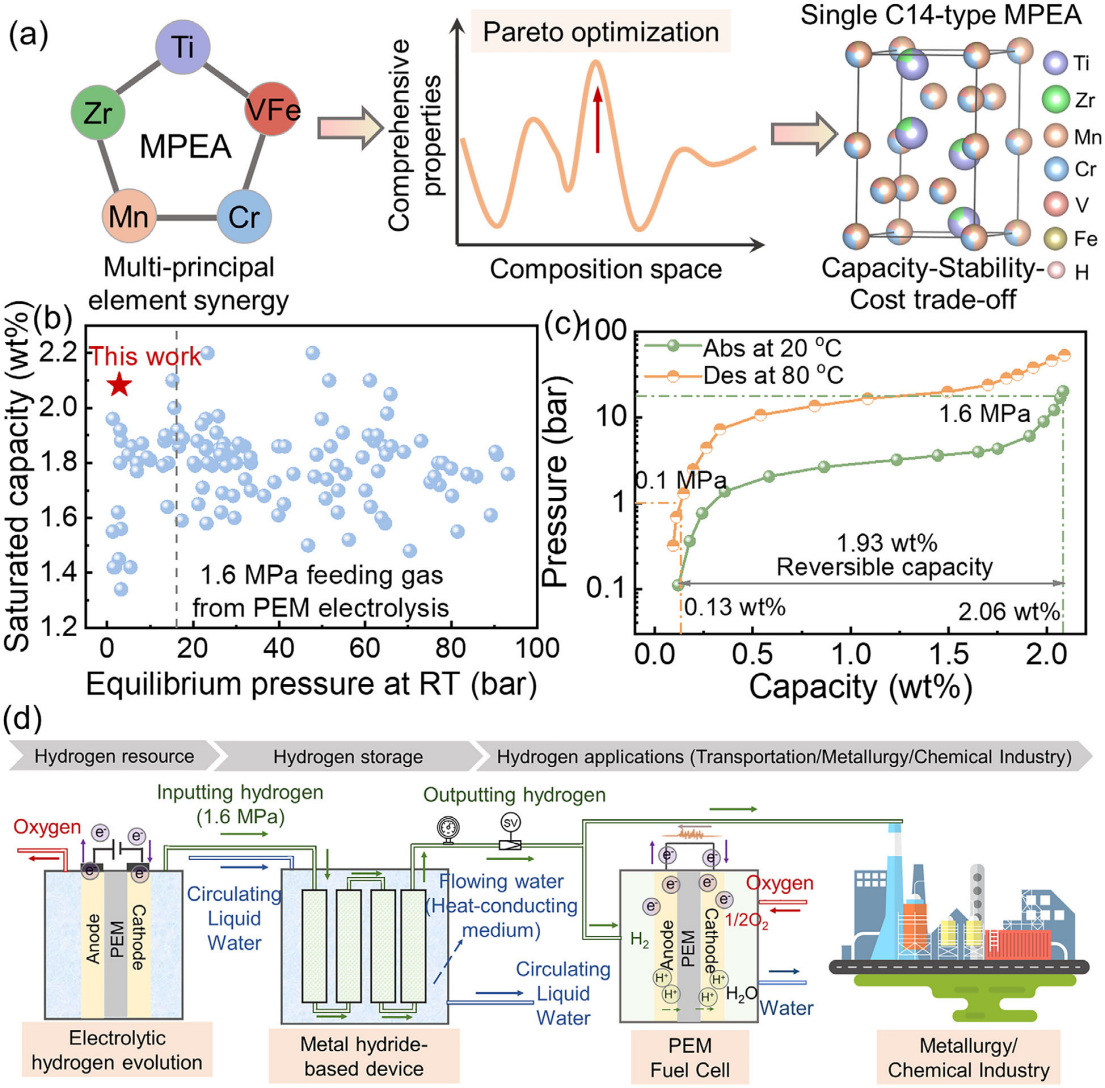
a) Design strategy adopted in this work. b) Comparison of room‐temperature hydrogen storage properties. c) De‐/hydrogenation properties of Ti_0.8_Zr_0.22_Mn_1.22_Cr_0.53_(VFe)_0.25_ MPEA at working condition. d) Schematic diagram of the application using electrolysis for hydrogen production as the hydrogen source and high‐density solid‐state HSMs as the energy storage medium.

Among the investigated MPEAs mentioned above, Ti_0.8_Zr_0.22_Mn_1.22_Cr_0.53_(VFe)_0.25_ MPEA emerges as the optimal composition, achieving 2.82 bar hydrogenation equilibrium pressure at 20 °C and 2.06 wt.% saturated hydrogen storage capacity at 1.6 MPa hydrogen pressure, surpassing the vast majority of known C14 Laves‐phase materials (**Figure** **3**a,b).^[^ ^20^, ^26^, ^27^, ^28^, ^29^, ^35^, ^41^, ^44^, ^45^, ^46^, ^47^, ^48^, ^49^, ^50^, ^51^, ^52^, ^53^, ^54^, ^55^, ^56^, ^57^, ^58^, ^59^, ^60^, ^61^, ^62^, ^63^ ^]^ Besides, the Ti_0.8_Zr_0.22_Mn_1.22_Cr_0.53_(VFe)_0.25_ MPEA demonstrates superior cost‐efficiency, as quantitatively evidenced in Figure S3 (Supporting Information), highlighting its strong potential for practical implementation. When heated to 80 °C, this MPEA demonstrates 1.93 wt.% reversible hydrogen storage capacity with 93.7% utilization efficiency (Figure 3c), confirming its suitability for low‐pressure hydrogen storage applications such as fuel‐cell‐based transportation, metallurgy, and chemical industry (Figure 3d).

### 2.4 Cycling Durability

**Figure 4:**
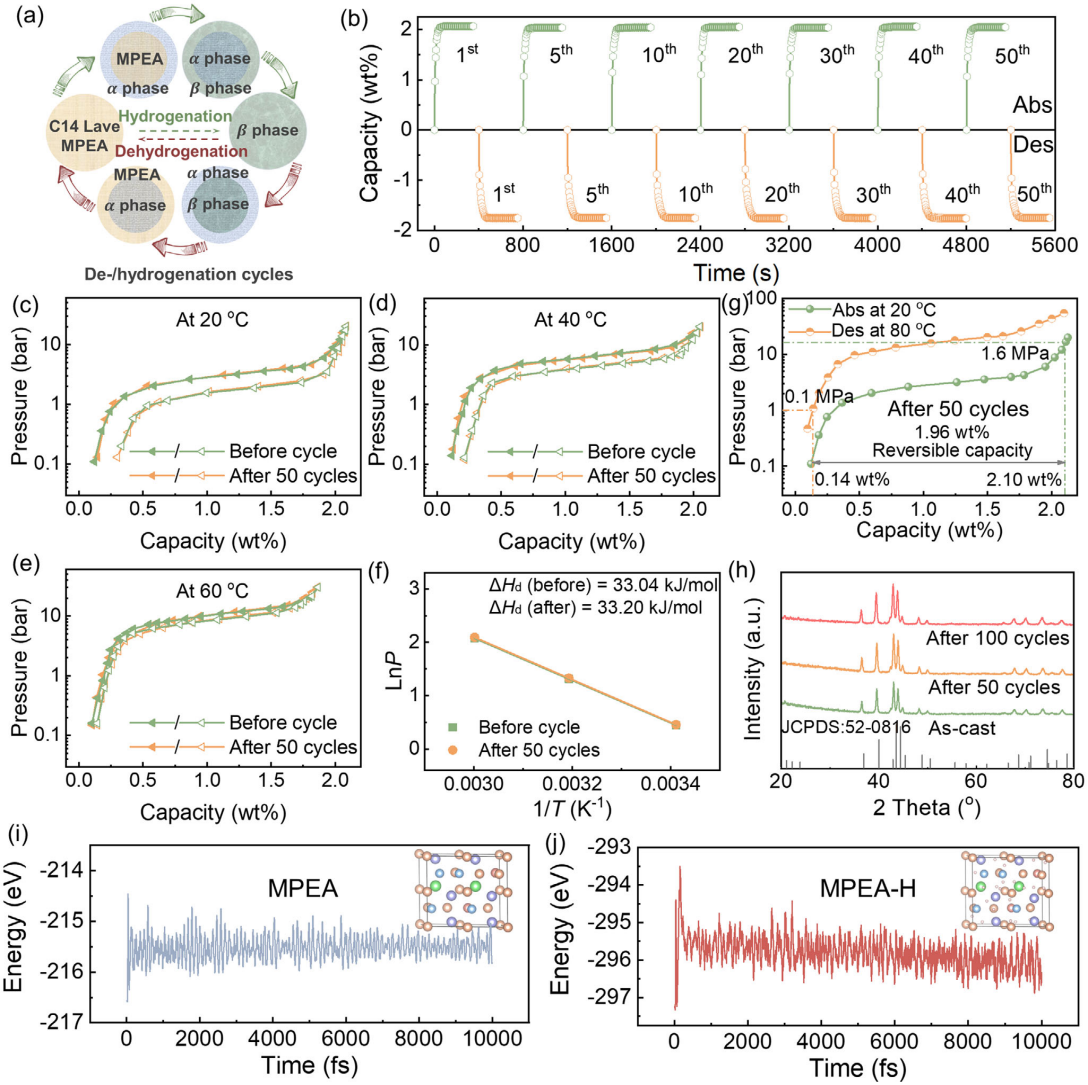
a) Schematic diagram of the reversible de‐/hydrogenation cycles. b) De‐/hydrogenation cycling kinetics, c–e) PCT curves, and f) Van't Hoff curve of Ti_0.80_Zr_0.22_Mn_1.22_Cr_0.53_(VFe)_0.25_ MPEA before and after 50 cycles. g) De‐/hydrogenation properties of Ti_0.80_Zr_0.22_Mn_1.22_Cr_0.53_(VFe)_0.25_ MPEA at working condition after 50 cycles. h) XRD patterns of Ti_0.80_Zr_0.22_Mn_1.22_Cr_0.53_(VFe)_0.25_ MPEA before and after cycling. Variation of total energy with time for i) MPEA and j) its hydride at 373.15 K.

The repeated insertion and extraction of hydrogen atoms in interstitial sites can induce lattice collapse, leading to deterioration in cycling stability. To introduce structural disturbance in Ti_0.8_Zr_0.22_Mn_1.22_Cr_0.53_(VFe)_0.25_ MPEA, we subjected it to rapid de‐/hydrogenation cycles (**Figure** **4**a). Specifically, hydrogenation was performed at 20 °C under 1.6 MPa H_2_ pressure, while dehydrogenation was carried out under dynamic vacuum at 100 °C.

Previous studies have demonstrated that hydrogen storage alloys are particularly prone to degradation during the initial 50 de‐/hydrogenation cycles, generally exhibiting disordered A/B‐side atomic occupancy (especially in interstitial metal hydrides) and irreversible phase disproportionation under practical operating conditions.^[^ ^41^, ^64^ ^]^ Accordingly, hydrogen storage kinetics were evaluated at periodic intervals (1st, 5th, 10th, 20th, 30th, 40th, and 50th cycles), with hydrogenation measured at 20 °C and dehydrogenation at an operational temperature of 60 °C. As shown in Figure 4b, Ti_0.8_Zr_0.22_Mn_1.22_Cr_0.53_(VFe)_0.25_ MPEA exhibits outstanding de‐/hydrogenation kinetics under working conditions. Specifically, it attains 90% of its saturated capacity within ≈20 s during hydrogenation and releases 90% of its reversible capacity in ≈30 s during dehydrogenation. Crucially, the kinetics demonstrate excellent cycling stability with no detectable capacity loss over 50 cycles. Further analysis via PCT measurements (Figure 4c–e), Van't Hoff fitting (Figure 4f) and operation properties (Figure 4g) confirm that the thermodynamic properties of this MEPA remain highly stable, showing no significant variation before and after 50 cycles. What's more, when extended the cycling tests to 100 cycles, there is still no degradation in de‐/hydrogenation capacity compared to the initial 50 cycles (Figure S4, Supporting Information), underscoring its potential for long‐term hydrogen storage applications. Compared with reported studies (Figure S3b, Supporting Information), the Ti_0.8_Zr_0.22_Mn_1.22_Cr_0.53_(VFe)_0.25_ MPEA in this work demonstrates superior performance in both saturated hydrogen storage capacity (2.06 wt.%) and cycling stability (100% retention). Based on comprehensive analysis, the multi‐objective optimization strategy developed herein successfully resolves the capacity‐stability‐cost trade‐off dilemma, and this MPEA exhibits outstanding potential as an efficient hydrogen storage medium for PEM water electrolysis systems.

**Figure 5:**
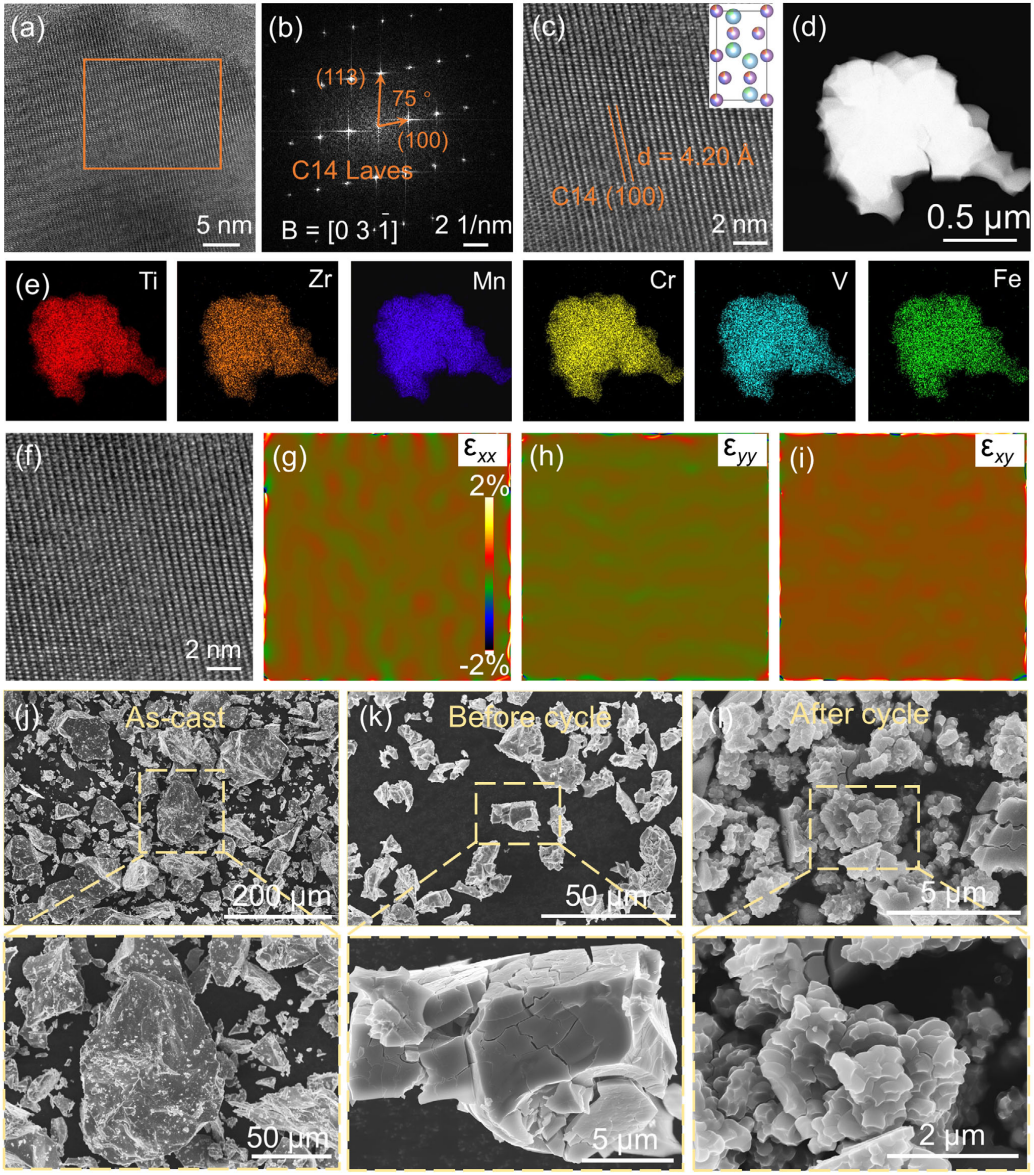
a) HRTEM image of after‐cycle Ti_0.80_Zr_0.22_Mn_1.22_Cr_0.53_(VFe)_0.25_ MPEA powders. b) FFT pattern and c) enlarged image of the yellowish‐brown region in Figure 5a. d) TEM bright field image as well as e) EDX mapping of after‐cycle Ti_0.80_Zr_0.22_Mn_1.22_Cr_0.53_(VFe)_0.25_ MPEA powders. f–i) Strain distributions of *x* (ε*_xx_*), *y* (ε*_yy_*), and *xy* (ε*_xy_*) directions for Ti_0.80_Zr_0.22_Mn_1.22_Cr_0.53_(VFe)_0.25_ MPEA powders after cycles. SEM images of Ti_0.80_Zr_0.22_Mn_1.22_Cr_0.53_(VFe)_0.25_ MPEA powders at j) as‐cast state, k) before‐cycle state, and l) after‐cycle state.

Phase structural stability is a critical technical parameter determining the practical applicability of hydrogen storage materials. XRD analysis in Figure 4h confirms that the MPEA maintains its C14 Laves single‐phase structure without secondary phase precipitation even after cycling. To further elucidate the fine‐scale microstructure, we conducted TEM characterization. The FFT pattern (**Figure** **5**b) corresponding to the orange region in Figure 5a exhibits distinct spots indexed to the (100) and (113) planes of the C14 Laves phase along the [031] zone axis. As shown in Figure 5c, the measured interplanar spacing of (100) planes are 4.20 Å, consistent with that of the as‐cast sample. During the de‐/hydrogenation processes, the repeated embedding and detachment of hydrogen atoms may lead to severe lattice distortion and localized strain after cycling. Therefore, we conducted geometric phase analysis (GPA) on the cycled (50 cycles) Ti_0.8_Zr_0.22_Mn_1.22_Cr_0.53_(VFe)_0.25_ MPEA. As shown in Figure 5f–h, the in‐plane strain distribution maps obtained via GPA from the HRTEM image of the same region are presented sequentially as ε*_xx_*, ε*_yy_*, and shear strain ε*_xy_*. The color scale is set to ± 2%, with green indicating negligible strain and red/blue representing tensile/compressive strain. Apart from the Fourier windowing effects at the edges, no significant strain concentration is observed within the field of view, indicating overall uniform lattice stress in this area.^[^ ^65^, ^66^, ^67^ ^]^ Besides, according to AIMD (Figure 4i,j), both the MPEA and its hydride exhibit excellent energy stability at 373.15 K (the highest temperature of liquid water), which is also consistent with the XRD and TEM results. Thus, these multi‐scale structural analyses collectively demonstrate exceptional cycling stability in phase structure.

Hydrogen‐induced pulverization phenomenon during activation and cycling is a common characteristic of all metal hydrides. Figure 5j–l presents the powder morphology of the as‐cast, activated dehydrogenated (before‐cycle), and after‐cycle (50 cycles) Ti_0.8_Zr_0.22_Mn_1.22_Cr_0.53_(VFe)_0.25_ MPEA, respectively. Notably, the activated MPEA exhibits numerous cracks propagating inward from the surface along with partially detached fine particles. After 50 and 100 cycles (Figure S7, Supporting Information), the surface morphology undergoes a significant transformation, developing a distinctive terraced hierarchical structure composed of multiple small facets, which aligns well with previous findings.^[^ ^41^, ^68^ ^]^ Thus, the localized strain generated by the repeated embedding and detachment of hydrogen atoms during the de‐/hydrogenation process can be promptly released through pulverization, thereby reducing the tendency of crystal structure destabilization and ensuring the stability of structural performance during cycling. Moreover, the homogeneous elemental distribution observed in the before‐cycle and after‐cycle samples (Figure 5e; Figures S5–S7, Supporting Information) across multiple length scales comprehensively demonstrates the exceptional cycling stability of constituent elements.

Theoretically, the de‐/hydrogenation process in hydrogen storage alloys inevitably induces lattice stress due to hydrogen insertion/extraction. When constituent elements occupy similar lattice sites, the alloy can only relieve this stress through continuous pulverization, which manifests as the observed pulverization phenomenon, a common characteristic of current material systems.^[^ ^20^, ^23^, ^35^, ^39^, ^41^, ^44^, ^69^, ^70^, ^71^ ^]^ Notably, unlike solid‐solution MPEAs where all atomic sites are equivalent (resulting in essentially random element distribution), intermetallic compounds feature specific site occupations:A‐side hydrogen‐absorbing elements predominantly occupy 4f sites while B‐side elements mainly occupy 2a and 6h sites in C14 Laves structure. Therefore, increasing the complexity of interstitial hydrogen storage environments requires incorporating more alloying elements. Consequently, developing such complex interstitial environments enables gradient stress transfer through neighbouring sites during de‐/hydrogenation cycles, fundamentally altering the stress relief mechanism and thereby reducing material pulverization rates. For a given material system, its intrinsic pulverization degree remains governed by fundamental physicochemical properties, where minor compositional modifications alone cannot achieve substantial improvements.

## 3 Conclusion

Through three sequential elemental screening steps and precise compositional engineering, this work achieves an optimal balance of chemical composition, geometric configuration, and hydrogen‐site binding energy. As a result, it successfully overcomes the capacity–stability–cost trilemma and leads to the development of a tailored Ti_0.8_Zr_0.22_Mn_1.22_Cr_0.53_(VFe)_0.25_ MPEA for hydrogen storage. This MPEA exhibits a hydrogenation equilibrium pressure of 2.82 bar at 20 °C and achieves a remarkable saturation capacity of 2.06 wt.% under 1.6 MPa hydrogen supply pressure, surpassing the vast majority of known C14 Laves‐phase materials. At 80 °C under 0.1 MPa hydrogen back pressure, it maintains a reversible hydrogen storage capacity of 1.93 wt.%, corresponding to an impressive 93.7% capacity utilization rate. Taking advantage of timely local strain release during repeated hydrogen insertion/extraction, this MPEA exhibits negligible deterioration in hydrogen storage properties, crystallographic structure, or elemental distribution throughout extended cycling. These outstanding characteristics provide a promising low‐energy, cost‐effective design strategy for high‐capacity, long‐cycle‐life solid‐state hydrogen storage applications.

## Conflict of Interest

The authors declare no conflict of financial interest.

## Supporting information



Supporting Information

## Data Availability

The data that support the findings of this study are available from the corresponding author upon reasonable request.
