# Supplementary material for: Resolving the Capacity‐Stability‐Cost Trilemma in Multi‐Principal‐Element Hydrogen Storage Alloys Through Multi‐Objective Optimization
Source: Adv Sci (Weinh). 2025 Aug 31;12(43):e13463. doi: 10.1002/advs.202513463 (PMC12631894; doi:10.1002/advs.202513463)
Supplement: Supplementary file 1 — Supporting Information [file ADVS-12-e13463-s001.docx]

***Supporting Information***

**Resolving the Capacity-Stability-Cost Trilemma in Multi-Principal-Element Hydrogen Storage Alloys through Multi-Objective Optimization**

Panpan Zhou ^b, c, 1^, Qianwen Zhou ^b, 1^, Wenzhe Liu ^b^, Nuo Lei ^b^, Yongpeng Chen ^b^, Jinghua Jiang ^c^, Dan Song ^c^, Hai-Wen Li ^a^, Qin-Yi Li ^e^, Lixin Chen ^b, d, *^, and Xuezhang Xiao ^a, b, *^

^a^ *School of Advanced Energy, Sun Yat-Sen University, Shenzhen 518107, Guangdong, China.*

^b^ *State Key Laboratory of Silicon Materials; School of Materials Science and Engineering, Zhejiang University, Hangzhou 310027, Zhejiang, China.*

^c^ *Jiangsu Provincial Engineering Research Center for Structure-Function Integrated Metallic Materials for Harsh Environments, College of Materials Science and Engineering, Hohai University, Changzhou 213200, Jiangsu, China.*

^d^ *Key Laboratory of Hydrogen Storage and Transportation Technology of Zhejiang Province, Hangzhou 310027, Zhejiang, China.*

^e^ *Department of Aeronautics and Astronautics, Kyushu University, Motooka744, Nishi-Ku, Fukuoka 819-0395, Japan.*

*^1^ These authors contributed equally.*

^*^Correspondence author.

*Email: lxchen@zju.edu.cn (L.X. Chen), xiaoxzh6@mail.sysu.edu.cn (X.Z. Xiao).*

**Experimental section**

***Materials Preparation***

In this work, high-purity (>99%) metallic raw materials (Ti, Zr, Mn, Cr, VFe) purchased from Beijing DM Material Technology Co., Ltd. were used without further purification. Specifically, two MPEA systems, Ti_0.85-_*_x_*Zr_0.17+_*_x_*Mn_1.2_Cr_0.55_(VFe)_0.25_ (*x* = 0, 0.05, 0.10) and Ti_0.80_Zr_0.22_Mn_1.2+_*_y_*Cr_0.55-_*_y_*(VFe)_0.25_ (*y* = 0, 0.02, 0.05), were synthesized via induction levitation melting (ILM). Firstly, the metallic raw materials were weighed according to the MPEA compositions, with an additional 3% Mn added to compensate for evaporation losses. Prior to melting, the raw materials were sequentially loaded into a water-cooled copper crucible in order of descending melting points, followed by five cycles of evacuation and argon purging to eliminate residual air in the chamber. The entire melting process, including heating and cooling, was conducted under a high-purity argon atmosphere. To ensure compositional homogeneity, each alloy ingot (approximately 30 g in weight) was flipped and remelted three times.

***Characterization***

Phase structure and crystallographic information of all investigated MPEAs were characterized by X-ray diffraction (XRD, X’Pert3 Powder-17005730) with Cu Kα radiation (40 kV×40 mA) and Rietveld refinement with Jade software, respectively. Actual composition was determined with inductively coupled plasma-optical emission spectrometry (Agilent 5110), and the pretreatment method of the sample was microwave digestion. Microstructural characterization was further performed using transmission electron microscopy (JEOL JEM F200, Japan) equipped with super energy-dispersive X-ray spectroscopy (JED 2300T) at 200 kV. Sample morphology and elemental distribution were analyzed using field-emission scanning electron microscopy (SEM, Hitachi SU8600) equipped with energy-dispersive X-ray spectroscopy (EDX). Strain analysis was conducted based on the geometric phase analysis (GPA) method^[1]^.

***Properties Measurements***

As-cast alloy ingots were first mechanically polished to remove surface oxides. Afterwards, the cleaned ingots were then pulverized into fine powders (<100 μm), and approximately 1.0 g powder was loaded into a stainless-steel reactor connected to a custom Sievert-type apparatus. The system was evacuated at room temperature for 1 hour to remove surface-adsorbed gases. Activation was achieved through multiple hydrogenation/dehydrogenation cycles using 5 MPa high-purity hydrogen (>99.99%). Following activation, pressure-composition-temperature (PCT) were conducted at 20 °C, 40 °C and 60 °C. Cycling properties were evaluated through repeated de-/hydrogenation to assess the stability of phase structure, elemental chemical states and hydrogen storage properties.

***Theoretical calculations***

In this study, we performed density functional theory (DFT) calculations using the vienna ab initio simulation package (VASP, Version 5.4.4) with the projector-augmented wave (PAW) method. The exchange-correlation effects were described within the generalized gradient approximation (GGA) using the Perdew-Burke-Ernzerhof (PBE) functional^[2-3]^. All calculations employed a plane-wave energy cutoff of 400 eV, with self-consistent convergence thresholds set to 0.01 eV/Å for ionic relaxation and 10^-6^ eV for electronic relaxation. To evaluate the hydride formation energy (Δ*E* = *E*(*A*_4_*B*_8_H_12_)-*E*(*A*_4_*B*_8_)-6**E*(H_2_), *A*: Hf, Nb, Y, Zr, Sc; *B*: V, Cr, VFe, Fe, Co, Ni, Cu, Mo), 20 different *AB*_2_H_3_ configurations with H atoms exclusively occupying tetrahedral sites were constructed with Python-based stochastic algorithm. Besides, to analysis the effects of Zr substitution on atomic hydrogen environments in lattice, we constructed a 2×1×1 HCP-type A_4_B_8_ supercell considering only the predominant B-side elements (Mn, Cr, V). Similarly, 50 distinct Ti_8_Mn_10_Cr_4_V_2_ configurations were generated and the lowest-energy structure was then selected ^[4-5]^. Since C14 Laves-phase hydrogen storage materials undergo an isostructural phase transformation process, the insertion/extraction of hydrogen atoms in interstitial sites only causes lattice expansion/contraction without altering the crystal structure. Subsequently, we constructed 50 Ti_8_Mn_10_Cr_4_V_2_H_24_ configurations with H atoms exclusively occupying tetrahedral sites and identified the most stable variant. This optimal structure then underwent systematic Zr substitution at A-side, producing 28 Ti_6_Zr_2_Mn_10_Cr_4_V_2_H_24_ configurations. Crystal orbital Hamilton population (COHP) analysis was performed with LOBSTER software to deeper insight of metal-H bonding ^[6-7]^. To evaluate the structure stability of both MPEA and its hydride, ab initio molecular dynamics (AIMD) simulations were performed from at 373.15 K in the NCT ensemble with Nose-Hoover thermostat (maximum duration: 10000 fs; time step: 1fs) ^[8-9]^.

***Statistics***

For hydrogen storage measurements, a Sieverts-type apparatus was used. The amount of absorbed/desorbed H_2_ was calculated using the gas equation (*PV* = *nRTz*) based on system pressure changes measured by a pressure sensor. In this gas equation, *P* is the pressure, *V* is the reactor/empty volume, *n* represents the change in the amount of hydrogen substance, *R* is the ideal gas constant (8.314 J/mol/K), *T* is the temperature, and *z* denotes the compressibility factor (*z* = 1 + α*P*/*T*, where α = 1.9155 × 10⁻^6^ K/Pa).

***Supplementary Figures***


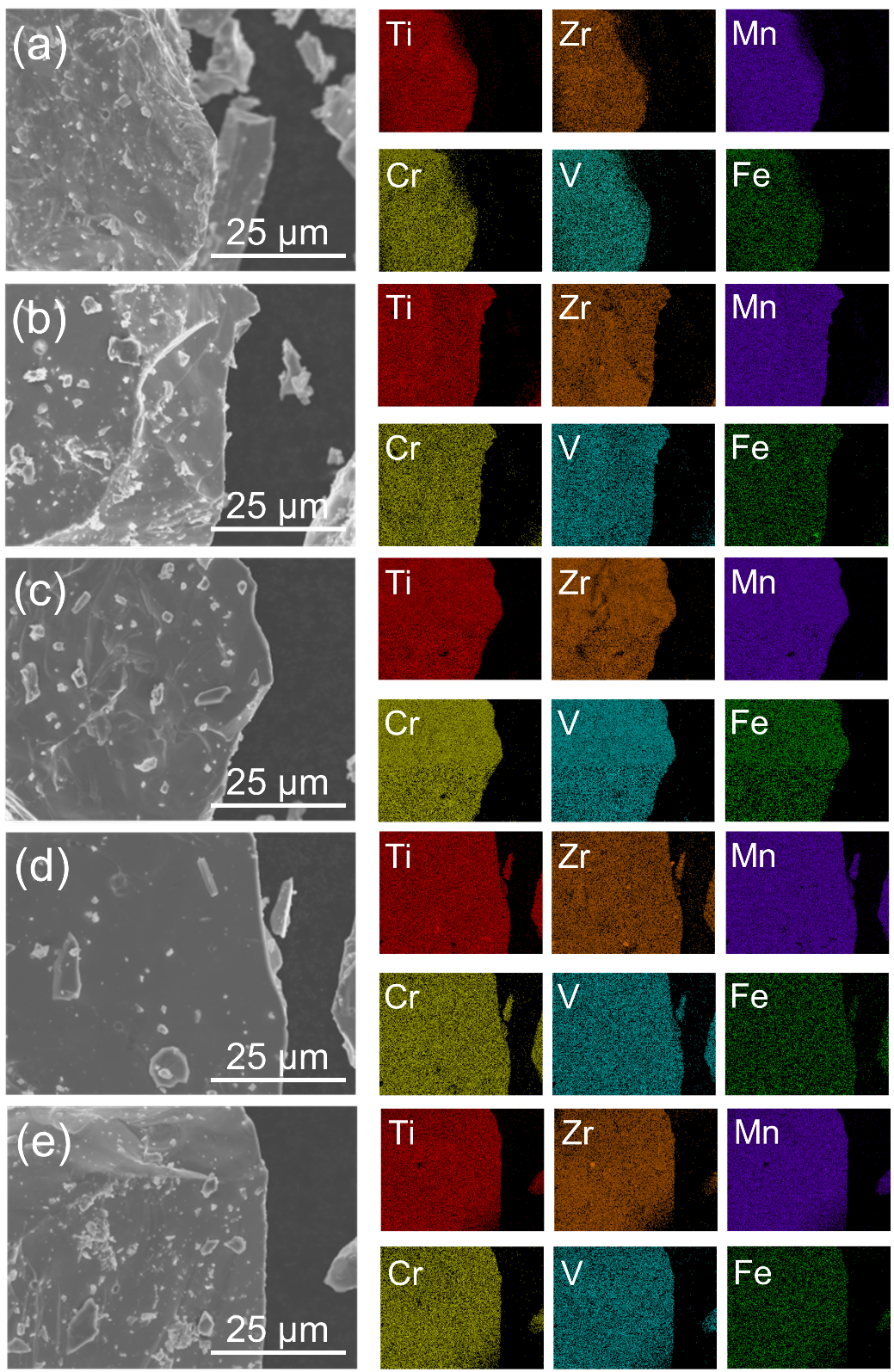


**Figure S1** SEM image and related EDX mappings of (a) Ti_0.85_Zr_0.17_Mn_1.2_Cr_0.55_(VFe)_0.25_, (b) Ti_0.8_Zr_0.22_Mn_1.2_Cr_0.55_(VFe)_0.25_, (c) Ti_0.75_Zr_0.27_Mn_1.2_Cr_0.55_(VFe)_0.25_, (d) Ti_0.8_Zr_0.22_Mn_1.22_Cr_0.53_(VFe)_0.25_ and (e) Ti_0.8_Zr_0.22_Mn_1.25_Cr_0.5_(VFe)_0.25_ MPEAs.


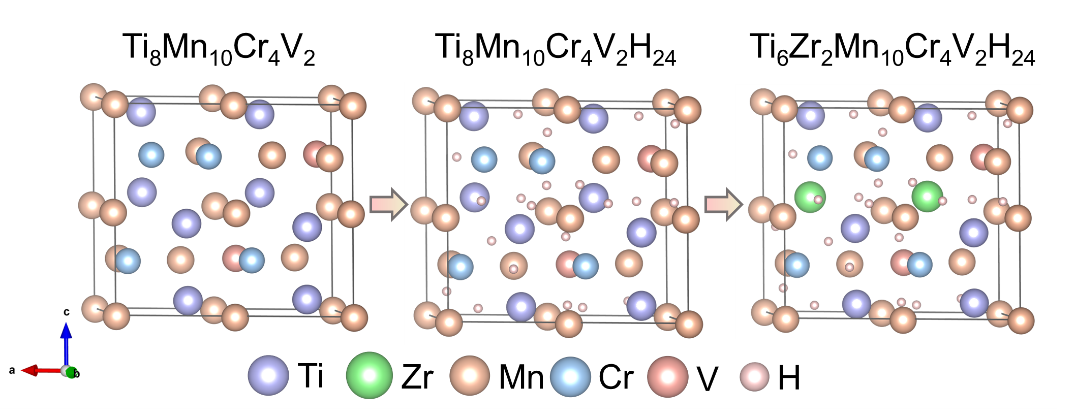


**Figure S2** Optimization processes and related configuration with the lowest energy.


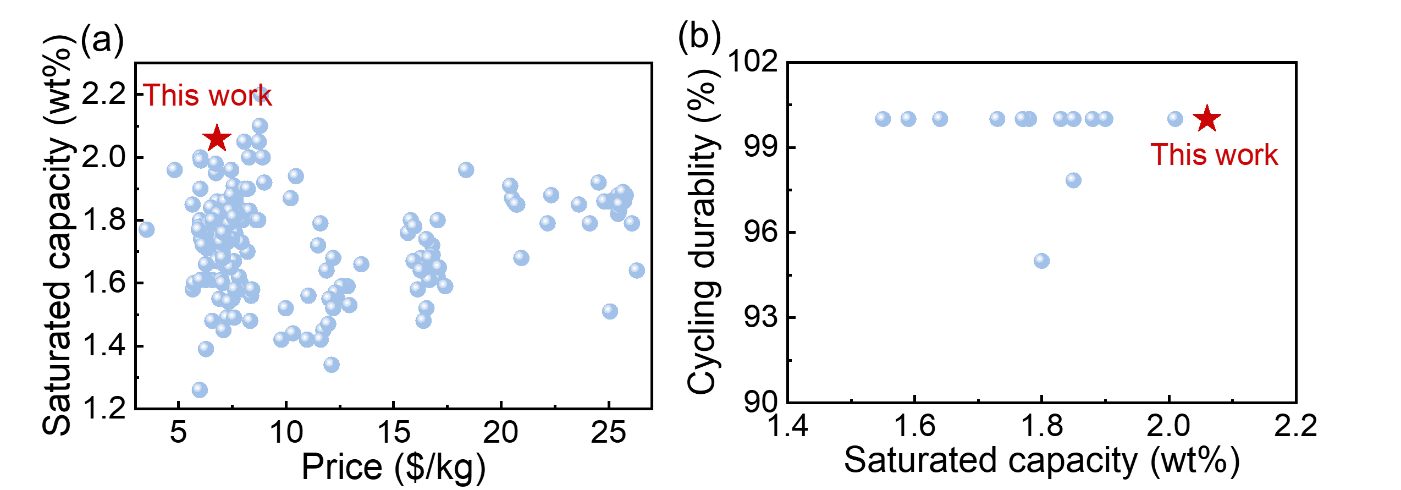


**Figure S3** Comparison of (a) cost-efficiency^[10-58]^ and (b) cycling durability^[14, 16-17, 34-35, 53-56, 58]^ for MPEA with C14 Laves structure.


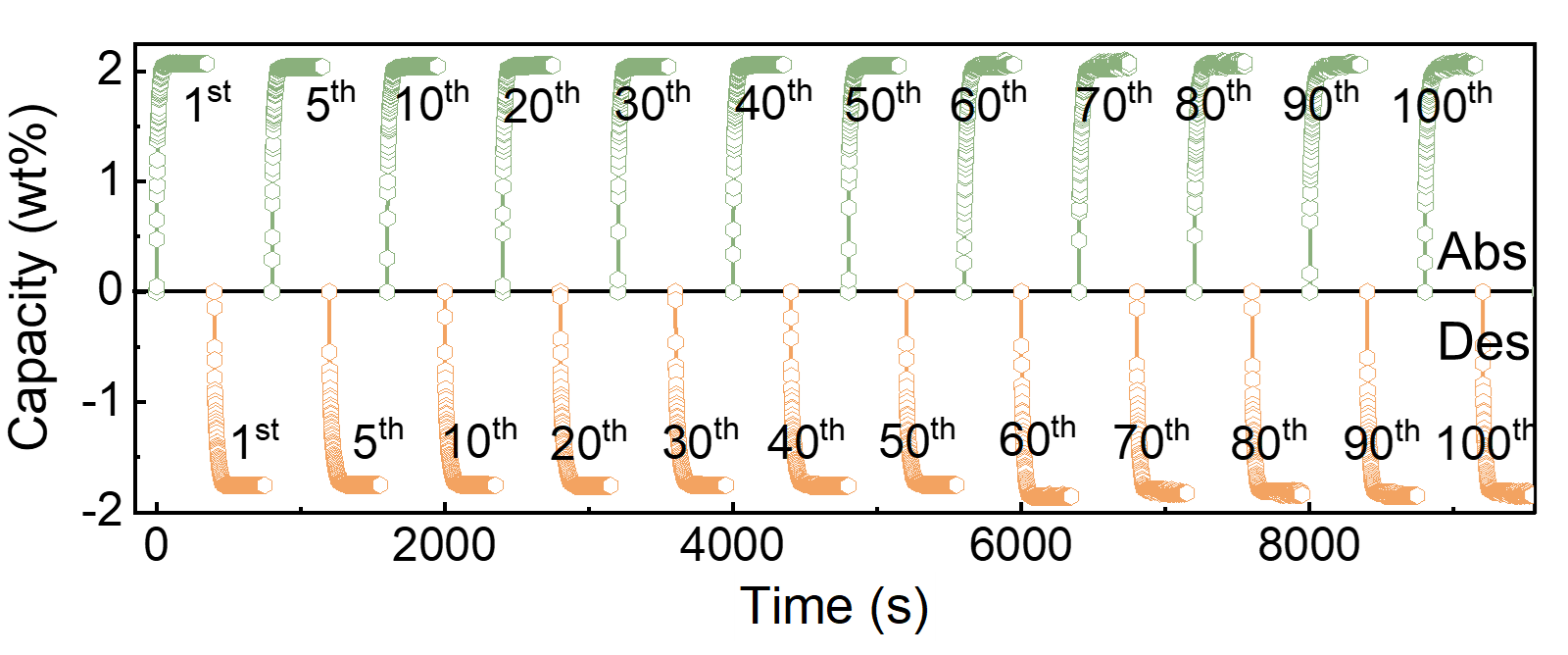


**Figure S4** De-/hydrogenation kinetics with extended cycles for Ti_0.80_Zr_0.22_Mn_1.22_Cr_0.53_(VFe)_0.25_ MPEA.


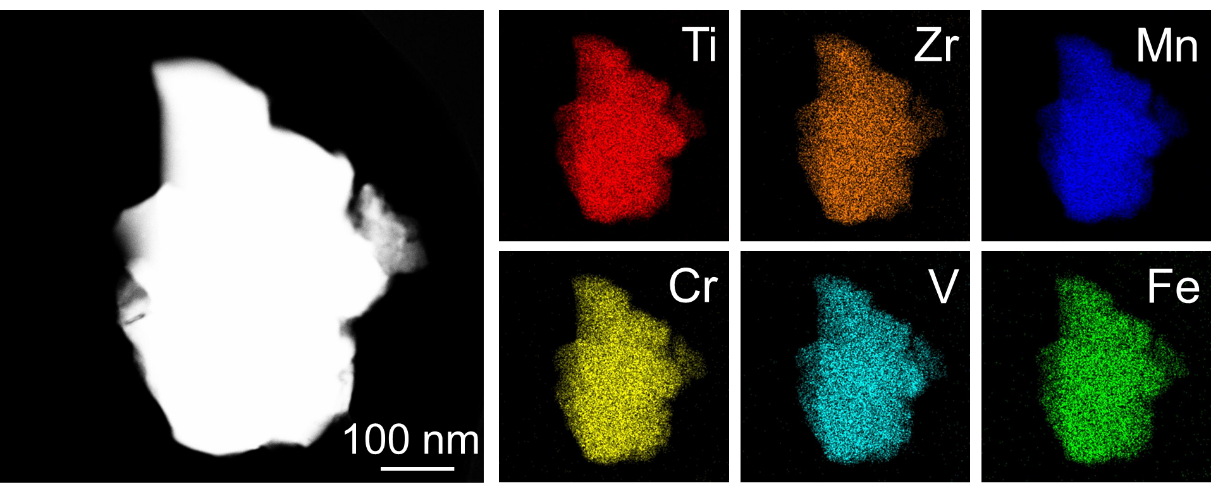


**Figure S5** TEM bright field image as well as EDX mapping of Ti_0.80_Zr_0.22_Mn_1.22_Cr_0.53_(VFe)_0.25_ MPEA powders before-cycle.


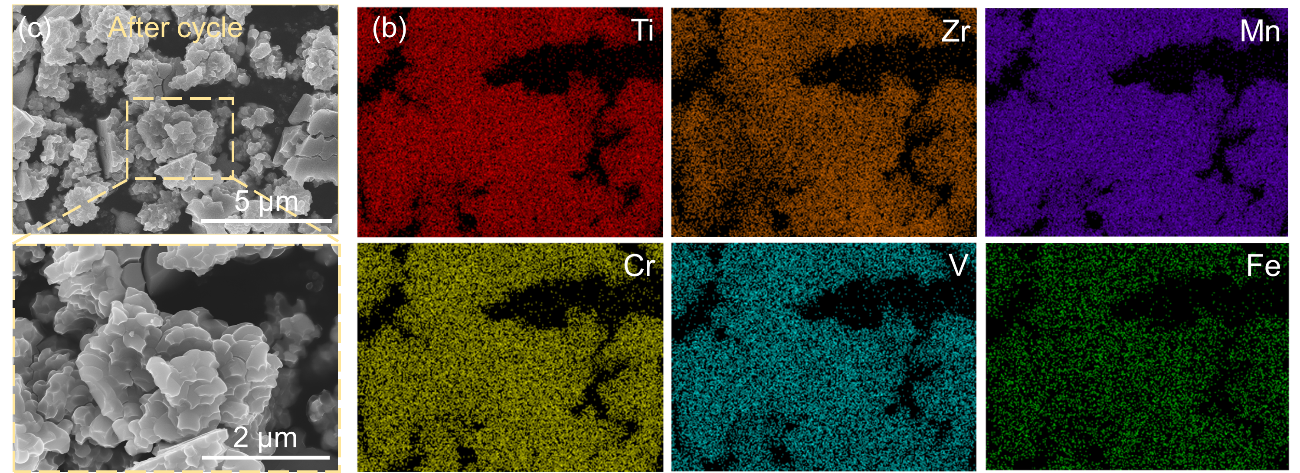


**Figure S6** (a) SEM image and (b) related EDX mappings of Ti_0.80_Zr_0.22_Mn_1.22_Cr_0.53_(VFe)_0.25_ MPEA after-50-cycles.


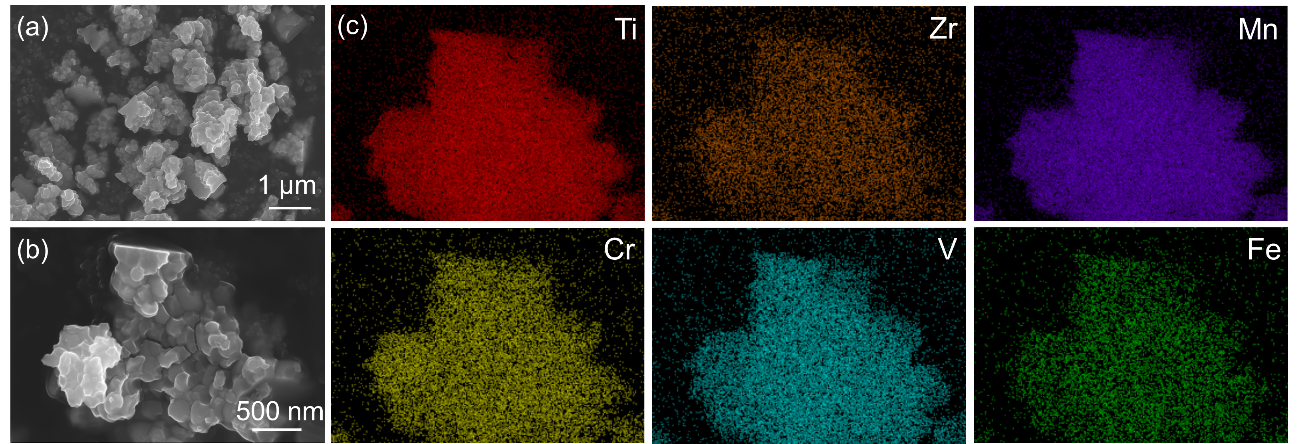


**Figure S7** (a-b) SEM image and (c) related EDX mappings of Ti_0.80_Zr_0.22_Mn_1.22_Cr_0.53_(VFe)_0.25_ MPEA after-100-cycles.

***Supplementary Tables***

**Table S1** Crystallographic information of Ti-Zr-Mn-Cr-(VFe)-based MPEAs.

| Alloy | ANOE | *r*_A_/*r*_B_ | Crystallographic parameters (Å) | | *a/c* | Cell volume(Å^3^) |
| --- | --- | --- | --- | --- | --- | --- |
|  |  |  | *a* | *c* |  |  |
| Ti_0.85_Zr_0.17_Mn_1.2_Cr_0.55_(VFe)_0.25_ | 5.686 | 1.161 | 4.8331 | 8.0819 | 0.5980 | 163.49 |
| Ti_0.8_Zr_0.22_Mn_1.2_Cr_0.55_(VFe)_0.25_ | 5.686 | 1.166 | 4.8520 | 8.1166 | 0.5978 | 165.48 |
| Ti_0.75_Zr_0.27_Mn_1.2_Cr_0.55_(VFe)_0.25_ | 5.686 | 1.171 | 4.8469 | 8.2358 | 0.5885 | 167.56 |
| Ti_0.8_Zr_0.22_Mn_1.22_Cr_0.53_(VFe)_0.25_ | 5.693 | 1.166 | 4.8457 | 8.1020 | 0.5981 | 164.75 |
| Ti_0.8_Zr_0.22_Mn_1.25_Cr_0.5_(VFe)_0.25_ | 5.703 | 1.166 | 4.8404 | 8.0920 | 0.5982 | 164.19 |

**Table S2** Designed and actual composition of Ti_0.8_Zr_0.22_Mn_1.22_Cr_0.53_(VFe)_0.25_ MPEA.

| Elements | Designed elemental content | Actual elemental content by ICP |
| --- | --- | --- |
| Ti | 23.08 wt% | 23.06 wt% |
| Zr | 12.10 wt% | 11.89 wt% |
| Mn | 40.40% wt% | 40.85 wt% |
| Cr | 16.61% wt% | 16.36 wt% |
| V | 6.22% wt% | 6.22 wt% |
| Fe | 1.60% wt% | 1.61 wt% |

**Reference**

[1] M. J. Hytch, E. Snoeck, R. Kilaas, *Ultramicroscopy* **1998**, 74, 131.

[2] J. P. Perdew, K. Burke, M. Ernzerhof, *Phys. Rev. Lett.* **1996**, 77, 3865.

[3] G. Kresse, D. Joubert, *Phys. Rev. B* **1999**, 59, 1758.

[4] J. Wang, P. Zhou, Y. Jia, J. Feng, J. Qi, F. Chu, L. Chen, X. Xiao, *Chem. Eng. J.* **2024**, 502, 157871.

[5] H. Shen, J. Hu, P. Li, G. Huang, J. Zhang, J. Zhang, Y. Mao, H. Xiao, X. Zhou, X. Zu, X. Long, S. Peng, *J. Mater. Sci. Technol.* **2020**, 55, 116.

[6] V. L. Deringer, A. L. Tchougréeff, R. Dronskowski, *J. Phys. Chem. A* **2011**, 115, 5461.

[7] R. Dronskowski, P. E. Blochl, *J. Phys. Chem.* **1993**, 97, 8617.

[8] J. Zhang, H. Zhang, S. Weng, R. Li, D. Lu, T. Deng, S. Zhang, L. Lv, J. Qi, X. Xiao, L. Fan, S. Geng, F. Wang, L. Chen, M. Noked, X. Wang, X. Fan, *Nat. Commun.* **2023**, 14, 2211.

[9] Y. Deng, X. Chen, H. Qi, S. Feng, W. Wang, L. Xie, G. Sun, H. Shen, X. Zu, H. Xiao, *Int. J. Hydrogen Energ.* **2024**, 87, 1327.

[10] E. Y. Anikina, V. N. Verbetsky, *Int. J. Hydrog. Energy* **2016**, 41, 11520.

[11] O. Beeri, D. Cohen, Z. Gavra, M. H. Mintz, *J. Alloys Compd.* **2003**, 352, 111.

[12] O. Bernauer, J. Topler, D. Noreus, R. Hempelmann, D. Richter, *Int. J. Hydrog. Energy* **1989**, 14, 187.

[13] Z. J. Cao, L. Z. Ouyang, H. Wang, J. W. Liu, D. L. Sun, Q. A. Zhang, M. Zhu, *Int. J. Hydrog. Energy* **2015**, 40, 2717.

[14] Z. J. Cao, L. Z. Ouyang, H. Wang, J. W. Liu, L. X. Sun, M. Felderhoff, M. Zhu, *Int. J. Hydrog. Energy* **2016**, 41, 11242.

[15] Z. J. Cao, L. Z. Ouyang, H. Wang, J. W. Liu, L. X. Sun, M. Zhu, *J. Alloys Compd.* **2015**, 639, 452.

[16] Z. M. Cao, M. Y. Piao, X. Z. Xiao, L. J. Zhan, P. P. Zhou, Z. N. Li, S. M. Wang, L. J. Jiang, F. Xu, L. X. Sun, L. X. Chen, *ACS Appl. Energy Mater.* **2023**, 6, 1913.

[17] Z. M. Cao, P. P. Zhou, X. Z. Xiao, L. J. Zhan, Z. F. Jiang, M. Y. Piao, S. M. Wang, L. J. Jiang, L. X. Chen, *J. Alloys Compd.* **2022**, 892, 162145.

[18] Z. M. Cao, P. P. Zhou, X. Z. Xiao, L. J. Zhan, Z. F. Jiang, S. M. Wang, L. J. Jiang, L. X. Chen, *Rare Metals* **2022**, 41, 2588.

[19] Z. M. Cao, P. P. Zhou, X. Z. Xiao, L. J. Zhan, Z. N. Li, S. M. Wang, L. X. Chen, *Int. J. Hydrog. Energy* **2021**, 46, 21580.

[20] V. Charbonnier, H. Enoki, K. Asano, H. Kim, K. Sakaki, *Int. J. Hydrog. Energy* **2021**, 46, 36369.

[21] Z. W. Chen, X. Z. Xiao, L. X. Chen, X. L. Fan, L. X. Liu, S. Q. Li, H. W. Ge, Q. D. Wang, *Int. J. Hydrog. Energy* **2013**, 38, 12803.

[22] Z. W. Chen, X. Z. Xiao, L. X. Chen, X. L. Fan, L. X. Liu, S. Q. Li, H. W. Ge, Q. D. Wang, *J. Alloys Compd.* **2014**, 585, 307.

[23] A. R. Galvis E, F. Leardini, J. Bodega, J. R. Ares, J. F. Fernandez, *Int. J. Hydrog. Energy* **2016**, 41, 9780.

[24] X. M. Guo, S. M. Wang, X. P. Liu, Z. N. Li, F. Lue, J. Mi, L. Hao, L. J. Jiang, *Rare Metals* **2011**, 30, 227.

[25] X. M. Guo, E. D. Wu, S. C. Wang, *Rare Metals* **2006**, 25, 218.

[26] M. T. Hagstrom, S. N. Klyamkin, E. V. Mescheryakova, P. D. Lund, *J. Mater. Sci.* **2000**, 35, 127.

[27] M. T. Hagstrom, J. P. Vanhanen, P. D. Lund, *J. Alloys Compd.* **1998**, 269, 288.

[28] D. G. Ivey, D. O. Northwood, *J. Mater. Sci.* **1983**, 18, 321.

[29] M. Kandavel, V. V. Bhat, A. Rougier, L. Aymard, G. A. Nazri, J. M. Tarascon, *Int. J. Hydrog. Energy* **2008**, 33, 3754.

[30] H. Li, X. H. Wang, Z. H. Dong, L. Xu, C. P. Chen, *J. Alloys Compd.* **2010**, 502, 503.

[31] J. Li, Y. Guo, X. Jiang, S. Li, X. Li, *Renew. Energy* **2020**, 153, 1140.

[32] J. G. Li, L. Xu, X. J. Jiang, X. G. Li, *Prog. Nat. Sci.-Mater.* **2018**, 28, 470.

[33] Q. Li, Z. Y. Peng, W. B. Jiang, L. Z. Ouyang, H. Wang, J. W. Liu, M. Zhu, *J. Alloys Compd.* **2021**, 889, 161629.

[34] Z. Y. Li, Y. H. Yan, H. X. Huang, B. G. Liu, Y. J. Lv, B. Zhang, W. Lv, J. G. Yuan, Y. Wu, *J. Alloys Compd.* **2022**, 908, 164605.

[35] H. Liu, L. Xu, X. Guo, Y. Wu, Z. Li, S. Wang, *Chin. J. Rare Met.* **2019**, 43, 928.

[36] P. Liu, X. Xie, L. Xu, X. Li, T. Liu, *Prog. Nat. Sci.* **2017**, 27, 652.

[37] K. Manickam, D. M. Grant, G. S. Walker, *Int. J. Hydrog. Energy* **2015**, 40, 16288.

[38] J. A. Murshidi, M. Paskevicius, D. A. Sheppard, C. E. Buckley, *Int. J. Hydrog. Energy* **2011**, 36, 7587.

[39] H. Oesterreicher, H. Bittner, *Mater. Res. Bull.* **1978**, 13, 83.

[40] Y. Osumi, H. Suzuki, A. Kato, K. Oguro, T. Sugioka, T. Fujita, *J. Less Common Met.* **1983**, 89, 257.

[41] J. M. Park, J. Y. Lee, *J. Less-Common Met.* **1990**, 160, 259.

[42] J. M. Park, J. Y. Lee, *J. Less-Common Met.* **1991**, 167, 245.

[43] Z. Y. Peng, Q. Li, J. Y. Sun, K. Chen, W. B. Jiang, H. Wang, J. W. Liu, L. Z. Ouyang, M. Zhu, *J. Alloys Compd.* **2022**, 891, 161791.

[44] F. Pourarian, W. E. Wallace, *J. Less-Common Met.* **1985**, 107, 69.

[45] J. Puszkiel, J. M. Bellosta von Colbe, J. Jepsen, S. V. Mitrokhin, E. Movlaev, V. Verbetsky, T. Klassen, *Energies* **2020**, 13, 2751.

[46] C. S. Qin, C. Zhou, L. Z. Ouyang, J. W. Liu, M. Zhu, T. Sun, H. Wang, *Int. J. Hydrog. Energy* **2020**, 45, 9836.

[47] V. M. Skripnyuk, M. Ron, *Int. J. Hydrog. Energy* **2003**, 28, 303.

[48] D. B. Smith, R. C. Bowman, L. M. Anovitz, C. Corgnale, M. Sulic, *J. Phys.: Energy* **2021**, 3, 034004.

[49] T. Z. Huang, Z. Wu, Y. X.B., C. J.Z., X. B.J., T. S. Huang, N. X. Xu, *Intermetallics* **2004**, 12, 91.

[50] B. Tu, H. Wang, Y. Wang, R. Li, L. Z. Ouyang, R. H. Tang, *Int. J. Hydrog. Energy* **2022**, 47, 14952.

[51] G. Y. Yu, F. Pourarian, W. E. Wallace, *J. Less-Common Met.* **1985**, 106, 79.

[52] T. A. Zotov, R. B. Sivov, E. A. Movlaev, S. V. Mitrokhin, V. N. Verbetsky, *J. Alloys Compd.* **2011**, 509, S839.

[53] P. P. Zhou, Z. M. Cao, X. Z. Xiao, L. J. Zhan, J. H. He, Y. Y. Zhao, L. Wang, M. Yan, Z. N. Li, L. X. Chen, *Mater. Today Energy* **2023**, 33, 101258.

[54] P. P. Zhou, Z. M. Cao, X. Z. Xiao, Z. F. Jiang, L. J. Zhan, Z. N. Li, L. J. Jiang, L. X. Chen, *Int. J. Hydrog. Energy* **2022**, 47, 1710.

[55] P. P. Zhou, Z. M. Cao, X. Z. Xiao, L. J. Zhan, S. Q. Li, Z. N. A. Li, L. J. Jiang, L. X. Chen, *J. Alloys Compd.* **2021**, 875, 160035.

[56] Y. Chen, Y. Jia, X. Xiao, Z. Cao, P. Zhou, L. Zhan, M. Piao, F. Chu, S. Yuan, L. Chen, *Int. J. Hydrog. Energy* **2024**, 86, 1376.

[57] M. Piao, X. Xiao, L. Zhan, Z. Cao, P. Zhou, J. Qi, M. Lu, Z. Li, L. Jiang, F. Fang, L. Chen, *Int. J. Hydrog. Energy* **2024**, 50, 1358.

[58] M. Piao, X. Xiao, Z. Cao, P. Zhou, L. Zhan, J. Qi, Z. Li, L. Jiang, L. Chen, *Mater. Chem. Phys.* **2023**, 297, 127407.
